# Development and Characterization of Bioactive Poly(butylene-succinate) Films Modified with Quercetin for Food Packaging Applications

**DOI:** 10.3390/polym13111798

**Published:** 2021-05-29

**Authors:** Łukasz Łopusiewicz, Magdalena Zdanowicz, Szymon Macieja, Krzysztof Kowalczyk, Artur Bartkowiak

**Affiliations:** 1 Center of Bioimmobilisation and Innovative Packaging Materials, Faculty of Food Sciences and Fisheries, West Pomeranian University of Technology Szczecin, Janickiego 35, 71-270 Szczecin, Poland; magdalena.zdanowicz@zut.edu.pl (M.Z.); szmacieja@gmail.com (S.M.); Artur-Bartkowiak@zut.edu.pl (A.B.); 2 Department of Chemical Organic Technology and Polymeric Materials, Faculty of Chemical Technology and Engineering, West Pomeranian University of Technology, Pułaskiego 10, 71-065 Szczecin, Poland; krzysztof.kowalczyk@zut.edu.pl

**Keywords:** quercetin, bioactive films, packaging films, antioxidant activity, poly(butylene succinate), PBS

## Abstract

The preparation of biodegradable active packaging materials is still a major challenge. Here, we report the fabrication and characterization of poly(butylene succinate)-based (PBS) films enriched with a natural polyphenolic antioxidant—quercetin. The PBS-based films with various quercetin content (0.05; 0.10; 0.25 and 0.50 pph on PBS) were prepared via a solvent casting method. Physical (optical, mechanical, thermal, moisture and water sorption, water vapor and UV–vis barrier) and biofunctional (antioxidant and antibacterial against *Escherichia coli* and *Staphylococcus aureus*) film properties were tested. The migration of quercetin into model food liquid systems was determined. As a result of quercetin addition, significant changes in color, opacity and UV-blocking effect were observed. The presence of the active substance did not significantly affect the thermal properties of the PBS matrix. However, the mechanical properties of the films were slightly decreased. The films exhibited excellent free radicals (DPPH, ABTS, O_2_^−^) scavenging and some bactericidal activities. PBS-quercetin films with superior functional properties have many possibilities for active food packaging applications.

## 1. Introduction

The packaging is likely the most important method for food preservation due to the fact that it protects, preserves and provides the needed information about the product while allowing product commercialization and distribution [1,2]. Nowadays, the food packaging industry strongly depends on the petrochemical non-biodegradable polymers. Their main advantages are wide applications, convenience, low prices and durability [1,2,3,4,5,6]. However, they have some disadvantages, such as association with other packaging materials and non-biodegradability, which makes them difficult to manage [2]. Based on available data, the annual plastics production exceeds 400 million tons, and around 40% is used for packaging purposes [7]. The increasing consumption of non-biodegradable plastics and large amount of waste material have led to severe deterioration and pollution of the environment as well as social problems [4,5,6,7,8,9]. Hence, both the recycling and biodegradability of plastics need serious attention [10,11]. Those issues have triggered extensive investigations to develop biodegradable polymers, especially derived from renewable resources [2,4,8,11].

In recent years, biodegradable polymers (such as biodegradable aliphatic polyesters) have gained worldwide attention both in fundamental research and industrial technologies because of their potential to minimize the environmental ballast caused by the disposal of non-biodegradable polymers [6,12]. Moreover, biopolymers have a great potential in biomedical and packaging applications [4]. A variety of biodegradable polymers have been tested, such as poly(lactic acid), polyhydroxyalkanoates, poly(ε-caprolactone), poly(butylene adipate-co-butylene terephthalate), and poly(butylene succinate) [3,4,6,8,12,13,14]. An interest in the mentioned polymers for food packaging applications has increased in the last two decades, posing one of the types of sustainable packaging materials. Together with biodegradable edible films (defined as a thin layer of biomaterial, such as polysaccharides, proteins, lipids, that can be consumed), they are considered optimal for future development in packaging technology, leading to the minimization of environmental pollution and exploitation of the world stock of fossil raw materials [2,3,5,12,15,16]. However, it is necessary to emphasize that innovative packaging materials should meet the criteria of laws that regulate the quality control of packaging in terms of their interaction with the food products (packaging–product relationship) [2]. Based on the Food and Drug Administration (FDA) requirements, the packaging should meet five basic requirements to be commercially available: (i) the packaging should not display any human health risk, (ii) the packaging should not change the physicochemical composition of the food, (iii) the packaging should not change the organoleptic features of the food, (iv) the packaging must be manufactured and treated according to good manufacturing practices, and (v) the packaging must not present misleading information about the contained product [1,2]. Other regulations established by the International Organization for Standardization (ISO 18604:2013(E)) as well as the European Union (such as regulation EC 10/2011 or regulation EC 1935/2004, which includes specific specifications on the use of active and intelligent packaging) should also be considered [1,2].

Among the biodegradable aliphatic polyesters, poly(butylene succinate) (PBS) has great potential for commercial applications due to its biodegradability, thermal stability, low melting point, relatively easy processibility in the industrial-scale manufacturing, and chemical resistance [3,4,6,8,10,13,14]. PBS is synthesized via a polycondensation process from succinic acid and 1,4-butanediol and consists of polymerized units of butylene succinate with repeating C_8_H_12_O_4_ units [3]. In recent years, the main feedstock to produce PBS, succinic acid, has been successfully derived from biobased resources [5,8,17]. Thus, it is identified as a safe and nontoxic potential material for biomedical and food packing applications [10,13,14]. However, poor impact strength, insufficient stiffness, low melt viscosity, as well as high production costs have limited its further applications [3,6,8,17]. The main production methods, such as chemical copolymerization, physical blending, and particle compounding, have been explored to improve these performances [6]. On the other hand, the low melting temperature of PBS can lower energy usage as well as prevent damage to other functional compounds and fillers, which can be added to improve various properties due to their burning or heating during processing [3]. In recent years, many attempts have been focused on developing PBS-based blends and composites with improved mechanical, thermal and gas-barrier properties as well as improved bioactivity (antioxidant and antimicrobial) at a lower cost by blending with natural polymers and inorganic nanoparticles [4,6,8,10,18,19,20]. The spherulitic morphology of PBS with different bio-fillers, such as cotton stalk bast fibers, wood flour, and bamboo fiber, was also investigated [10].

The problem of food loss due to spoilage by microorganisms as well as unfavorable changes due to oxidation can be addressed by using active packaging [1,2]. In recent years, different types of active compounds have been incorporated into food biodegradable packaging films to enhance their physicochemical properties as well as antioxidant and antimicrobial ability [20,21,22]. The development of antimicrobial packages is a promising path for active control of bacterial and fungal proliferation that leads to food spoilage. Antimicrobial packages have a crucial role in food safety and preservation. This type of package increases the latency phase and reduces the growth of microorganisms, enhancing the food quality and safety, ensuring a longer shelf life. By using antimicrobial materials for food packaging, the shelf life is extended and the growth of microorganisms is slowed, thus ensuring better quality and safety of food products [1]. Antioxidants are widely used as food additives to improve the oxidation stability of sensitive foods and to prolong a product shelf-life [23]. Synthetic phenolic antioxidants, including butylated hydroxytoluene (BHT), butylated hydroxyanisole (BHA), as well as tertiary butylhydroquinone (TBHQ), are commonly used because of their chemical stability, low cost, and availability. However, the safety of these synthetic compounds has been questioned [20,24]. There is much interest among food manufacturers in using natural antioxidants as replacements for the synthetic antioxidants that are currently being used. Moreover, the addition of active agents in the polymeric matrix can affect the mechanical, thermal, optical and gas barrier properties of the packaging materials [9,20,23,25,26]. A wide spectrum of additives (nano or microsized) has been used for the preparation of active films [1,2,12]. The methods employed in obtaining antioxidant and antimicrobial activity vary from adding sachets with volatile active agents inside current packaging to incorporating the active agent directly into biopolymers and from coating or grafting active compounds on the polymeric surface for the use of intrinsic active pads [1]. In the last two decades, especially due to the consumer’s demands, there has been an increasing interest in the incorporation of natural antioxidants in the active packaging. In this sense, natural polyphenolic compounds have received considerable attention due to their high antioxidant activity [20,23]. Polyphenols belong to a wide category of plant-derived natural compounds exhibiting many biological properties, including anticancer, anti-inflammatory, antimicrobial and antioxidant activity [27]. The application of phenolic compounds and phenolic extracts in active packaging has attracted a particular interest since these compounds show potent antimicrobial and antioxidant activity in food systems and their intake can make a contribution to human health [9,27]. The successful incorporation of numerous phenolic compounds as well as phenolic-rich extracts into biodegradable films (including edible films) has been reported [28,29,30,31,32]. As the phenolic compounds are able to interact with the environment and the product to extend its shelf life, their addition to the packaging material reduces or even eliminates the need to use synthetic antioxidants [2]. Thus, the risk of potential toxicity caused by molecule migration is limited, at the same time protecting the contents of the package [27].

Quercetin (3,3′,4′,5,7-Pentahydroxyflavone) is considered one of the most common dietary phenolic compounds widely distributed in the plant kingdom, being a crucial component of tea, fruits and vegetables (onions, apples, berries, cherries, broccoli) as well as wine [21,33,34]. This polyphenol has been demonstrated to exhibit strong antioxidant, anti-inflammatory, anticarcinogenic, and antiviral properties, acting as a free-radical scavenger or metal ion chelating agent [34]. Moreover, quercetin is commonly used as an additive in cosmetics and food to prevent the oxidative processes responsible for product deterioration [27]. Thus, the incorporation of quercetin into biopolymer-based films can greatly enhance the antioxidant ability of the films, but it has, comparatively, been rarely investigated as a component of the active food packaging film ingredient to date. However, quercetin has been successfully incorporated into several different films, such as zein, chitosan, chitosan–gelatin, cassava starch–carboxymethyl cellulose, cellulose, kafirin, ethylene vinyl alcohol, poly(vinyl alcohol), ethylene vinyl acetate, low-density polyethylene, polypropylene and carboxymethyl cellulose films (including films used for food packaging applications) [21,22,23,24,27,34,35,36,37,38,39]. Nevertheless, there is no study related to utilizing quercetin as an additive for PBS films aimed to provide antioxidant active food packaging materials.

This study aims to fabricate PBS-based films with quercetin addition as a potential material for food packaging. PBS-quercetin films were prepared and characterized using various techniques: mechanical tests, UV–vis (UV-visible) and FT-IR (Fourier transform infrared) spectroscopy, DSC (differential scanning calorimetry) and TGA (thermogravimetric analysis) measurements as well as WVTR (water vapor transmission rate) determination. Furthermore, their physical and biological properties were determined. Their potential for antimicrobial and antioxidant food packaging applications is discussed.

## 2. Materials and Methods

### 2.1. Materials and Reagents

Poly(butylene succinate)—PBS (FZ91PM BioPBS™) was purchased from Mitsubishi Chemical (Tokyo, Japan). Quercetin, calcium chloride, sodium chloride, hydrogen peroxide, disodium phosphate, monosodium phosphate, 2,2-diphenyl-1-picrylhydrazyl (DPPH), 2,2′-azino-bis(3-ethylbenzothiazoline-6-sulfonic acid) (ABTS), potassium persulphate, potassium ferricyanide, trichloroacetic acid, ferric chloride, iron sulphate, tris(hydroxymethyl)aminomethane and pyrogallol were purchased from Merck (Darmstadt, Germany). Hydrochloric acid, acetic acid, sodium hydroxide, chloroform, ethanol and methanol were supplied from Chempur (Piekary Śląskie, Poland). Mueller-Hinton broth and Mueller-Hinton agar were purchased from Merck (Darmstadt, Germany). All chemicals were of analytical grade. Escherichia coli ATCC25922 and Staphylococcus aureus ATCC43300 were purchased from ATCC (Manassas, VA, USA).

### 2.2. Preparation of Films

PBS, quercetin—Q (100/0.05; 100/0.10; 100/0.25 and 100/0.50 PBS/Q weight fraction) and chloroform were placed in tightly closed glass bottles and stirred on a magnetic stirrer (250 rpm) until total dissolution of PBS. Then, systems were poured (10 g) onto glass Petri dishes (90 mm diameter) and dried at 45 °C for 24 h. The dried films were peeled off from the plates and were conditioned for 3 days at 25 °C and 50% RH in a climate room prior to any tests. The acronyms for the samples are PBS-QX, where X is a fraction of Q per 100 parts of PBS.

### 2.3. Thickness and Mechanical Properties of the Films

Film thickness was measured with a digital micrometer (Dial Thickness Gauge 7301, Mitoyuto Corporation, Kanagawa, Japan, with an accuracy of 0.001 mm), through measurements at ten random locations around each film sample and expressed as average ± standard deviation [34]. The average values were used in the calculation of water vapor transmission rate and mechanical properties. Mechanical tests were performed using a Zwick/Roell Z2.5 tensile tester (Ulm, Germany) according to ASTM D822-02. The films were cut into 10 mm wide strips, the initial grip separation was 25 mm and the cross-head speed was 10 mm/min. At least six replicate samples were tested. The elongation at break (EB), maximum tensile strength (TS), Young’s modulus (YM) with standard deviations were calculated by TestXpert II software.

### 2.4. The Water Vapor Transmission Rate of the Films

Gravimetricaly methodology was used for determination of the water vapor transmission rate (WVTR) of films, applying the procedure described in a previous study [25]. Initially, the amount of dry CaCl_2_ inside the container was 9 g. The area of film samples was 8.86 cm^2^. Measurements were carried out for a period of 4 days, and each day the containers were weighed to determine the amount of absorbed water vapor through the films (stored at RH 80% and 25 °C). The results were expressed as average values from each day of measurement and each container. Analyses were carried out at 10 independent containers (10 repetitions) for each type films, calculated as a standard unit g/(m^2^ × day) and presented as a mean ± standard deviation.

### 2.5. UV–Vis and FT-IR Spectroscopic Analysis

The UV–Vis spectra (transmittance and absorbance in a region 200–800 nm) of the film samples were measured using a UV–Vis Thermo Scientific Evolution 220 spectrophotometer (Waltham, MA, USA). Transmission infrared spectra of the films were measured at room temperature using a Perkin Elmer Spectrum 100 FT-IR spectrometer (Waltham, MA, USA) in the range 4000–650 cm^−1^ with 62 scans, 1 cm^−1^ resolution. The films were placed in the sample holder. For analysis, spectra were baseline corrected and normalized using SPECTRUM software.

### 2.6. Moisture Content, Moisture Sorption and Water Sorption Degrees

To determine moisture content (MC) of the films, the samples were dried at 65 °C for 24 h, and the weight change was analyzed [25]. For moisture sorption (MS) and water sorption (WS), PBS-based films samples 20 mm × 20 mm were prepared. Samples were placed in a vacuum dryer (250 millibar) at 65 °C for 24 h. The mass of the dried samples, stored in a climate chamber and kept at 25 °C/50% and 80% RH (for moisture sorption evaluation) or immersed in distilled water for 24 h (for swelling behavior investigation), were determined [40].

### 2.7. Thermal Characterization

Thermogravimetric analysis—TGA (Q2500, TA Instruments Inc., New Castle, DE, USA) for the films was performed using a platinum pan under 25 mL/min air flow, in the temperature range 40–900 °C at a heating rate 10 °C/min. Differential scanning calorimetry (DSC) analysis was performed with Q100 DSC (TA Instruments Inc., New Castle, DE, USA). Selected samples (PBS, PBS-Q0,10 and PBS-Q0.50) were analyzed under nitrogen atmosphere, in the heating–cooling–heating cycle, with samples weighing approximately 10 mg. The temperature range cycles were as follows: first heating from 0 to 150 °C, cooling from 150 to −70 °C and second heating from −70 to 350 °C with a heating/cooling rate of 20 °C/min.

### 2.8. Color and Opacity Measurements

Colorimeter (CR-5, Konica Minolta, Tokyo, Japan) was used for color determination using the CIELab color scale. Samples were analyzed in 10 repetitions, taken at random locations on each of the studied films. The total color difference (Δ*E*), yellowness index (*YI*) and chroma (*C*) were calculated according the following formulas [25]:

$$\Delta E={\left[{\left(L_{standard\;}-L_{sample}\right)}^{2\;}+{\left(a_{standard}-a_{sample}\right)}^{2}+{\left(b_{standard}-b_{sample}\right)}^{2}\right]}^{0.5}$$

$$YI=142.86\times b\times L^{-1}$$

$$C=arctg\frac{b_{sample}}{a_{sample}}$$

The opacity of modified PBS films and pure PBS was determined in an Opacimeter EE Model 12 (Diffusion Systems LTD). The opacimeter was initially calibrated using a standard white plate (value 100 ± 1, Diffusion Systems, LTD), and measurements were performed on each film six times and presented as mean ± standard deviation [12].

### 2.9. Reducing Power and Free Radicals Scavenging Activity

Antixodiant activity (as a capacity to scavenge free radicals—DPPH, ABTS and O_2_^−^) and reducing power of PBS-based films were determined by using spectroscopic methods according to the procedures described elsewhere [25]. The DPPH radical scavenging activity was determined by placing 100 mg of each film in 25 mL of 0.01 mM DPPH methanolic solution, incubated for 30 min at room temperature. Then, the absorbance was measured at 517 nm. To determine ABTS radical scavenging activity, 10 mL of ABTS solution was mixed with 100 mg of the films and the absorbance was measured at 734 nm after 6 min of incubation. The O_2_^−^ radical scavenging activity was analyzed by mixing 3 mL of 50 mmol/L (pH 8.2) Tris-HCl buffer with 100 mg of the films. Then, a pyrogallol solution (0.3 mL, 7 mmol/L, preheated to 25 °C) was added, and allowed to react for exactly 4 min. Finally, 1 mL od 10 mmol/L of HCl was added to terminate the reaction, and absorbance was measured at 318 nm. The reducing power was determined by placing the film samples (100 mg) in 1.25 mL of phosphate buffer (0.2 M, pH 6.6) followed by the addition of 1.25 mL of 1% potassium ferricyanide solution. Subsequently, the samples were incubated for 20 min at 50 °C followed by the addition of 1.25 mL of trichloroacetic acid. Then, the test tubes were centrifuged at 3000 rpm for 10 min. The obtained supernatant (1.25 mL) was diluted with 1.25 mL of deionized water. Finally, 0.25 mL of 0.1% ferric chloride solution was added and the absorbance was measured at 700 nm.

### 2.10. Antibacterial Activity

The film samples were cut into square shapes (3 × 3 cm) and their antibacterial activity was evaluated based on ASTM E 2180-01 methodology with the modification described in a previous study [25]. *E. coli* and *S. aureus* cultures originated from 24 h growth (coming from stock cultures) were firstly prepared. The concentrations of the cultures were standardized to 1.5 × 10^8^ CFU/mL. The concentration of each culture was measured using a Cell Density Meter (WPA, Cambridge, UK. CB4 OF J). The agar slurry was prepared by dissolving 0.85 g of NaCl and 0.3 g of agar–agar in 100 mL of deionized water and autoclaved for 15 min at 121 °C and equilibrated at 45 °C (one agar slurry was prepared for each strain). One milliliter of the culture (separately) was placed into the 100 mL of agar slurry. The final concentration of each culture was 1.5 × 10^6^ CFU/mL in molten agar slurry. The square samples of each film were introduced (separately) into the sterile Petri dishes with a diameter of 55 mm. Inoculated agar slurry (1.0 mL) was pipetted onto each square sample. The samples were incubated for 24 h at 30 °C with relative humidity at 90%. After incubation, the samples were aseptically removed from the Petri dishes and introduced into the 100 mL of the Mueller-Hinton Broth. The samples were dispersed for 1 min in the Bag Mixer^®^ CC (Interscience, St Nom la Brètech, France). The dispersion facilitated the complete release of the agar slurry from the samples. Then serial dilutions of the initial inoculum were performed. Each dilution was spread into the Mueller-Hinton Agar and incubated at 37 °C for 24 h. The results were presented as an average value with standard deviations.

### 2.11. Determination of Quercetin Migration into Model Food Liquid Systems

The migration of quercetin into model food liquid systems was determined based on the methodology of Łupina et al. [41] with a slight modification. The model food liquid systems were selected taking into account the European Union Commission Regulation (2020/1245) on plastic materials and articles intended to come into contact with food and were as follows: distilled water, 96% ethanol, 50% ethanol, 20% ethanol, 10% ethanol, 3% acetic acid, and 0.01 M NaOH. The PBS-based films specimens (2 × 2 cm) were shaken with liquids (25 mL, 25 ± 1 °C, 120 rpm, 24 h) in the shaking incubator. Two hundred and fifty microliters of the release media samples were taken at different time points (0, 15, 30, 45, 60, 120, 180 min and 24 h) and the absorbance was read at 370 nm using a microplate spectrophotometer (Synergy LX Microplate Spectrophotometer, BioTek, Winooski, VT, USA).

### 2.12. Statistical Analysis

Statistical analyses were carried out using Statistica version 10 (StatSoft Polska, Kraków, Poland). Differences between means were determined by Fisher’s LSD post hoc testing with a significance threshold of *p* < 0.05.

## 3. Results and Discussion

### 3.1. Free Radicals Scavenging Activity and Reducing Power

The antioxidant activity of the PBS-based films was evaluated by free radicals (DPPH, ABTS, O_2_^−^) scavenging methods as well as reducing power measurements, and the final results are shown in Table 1. As expected, the neat PBS film showed almost negligible activity against free radicals, which is consistent with the results reported by Domínguez-Robles et al. [42]. Conversely, the quercetin-enriched films showed a significant increase of antioxidant activity and reducing power (*p* < 0.05) due to the retained ability of phenolic groups of quercetin to donate hydrogen to stabilize free radicals [23,34,39]. Moreover, a dose-dependent increment was observed. It is readily established that biopolymer-based films’ antioxidant activity is proportional to the antioxidant additives content [9,25,26]. The highest antioxidant activity (80.90 ± 0.07%, 99.07 ± 0.28%, 38.06 ± 0.11%, for DPPH, ABTS and O_2_^−^ radicals, respectively) and reducing power (2.08 ± 0.01) was noticed for sample PBS-Q0.50. These findings are consistent with the results of other authors reported high antioxidant activity of quercetin-modified films [21,22,23,27]. On the other hand, Tongdeesoontorn et al. reported that the DPPH scavenging activity of cassava starch–carboxymethyl cellulose (CMC) films did not change with the increase of quercetin concentration [24]. Among the flavonoids, quercetin is one of the most effective antioxidants due to the *o*-hydroxy structure in the B ring, the 2,3 double bond in conjugation with 4-oxo function in the C-ring and to 3- and 5-OH groups with the 4-oxo function in the A and C rings [34]. The high antioxidant activity of PBS films with quercetin indicated their potential as an additive for active, functional packaging systems for prevention of oxidation-sensitive food matrices, as well as prolongation their shelf-life [9,25,43]. This is of particular importance, as quercetin has the ability to intercept free radical chains, inducing the formation of stable end products that do not initiate or propagate oxidatively sensitive compounds, such as lipids [27]. In fact, the application of starch-CMC films incorporated with quercetin as active food packaging was reported [36]. However, further tests on model food products for PBS-quercetin films should be carried out.

### 3.2. Antibacterial Activity

**Figure 1:**
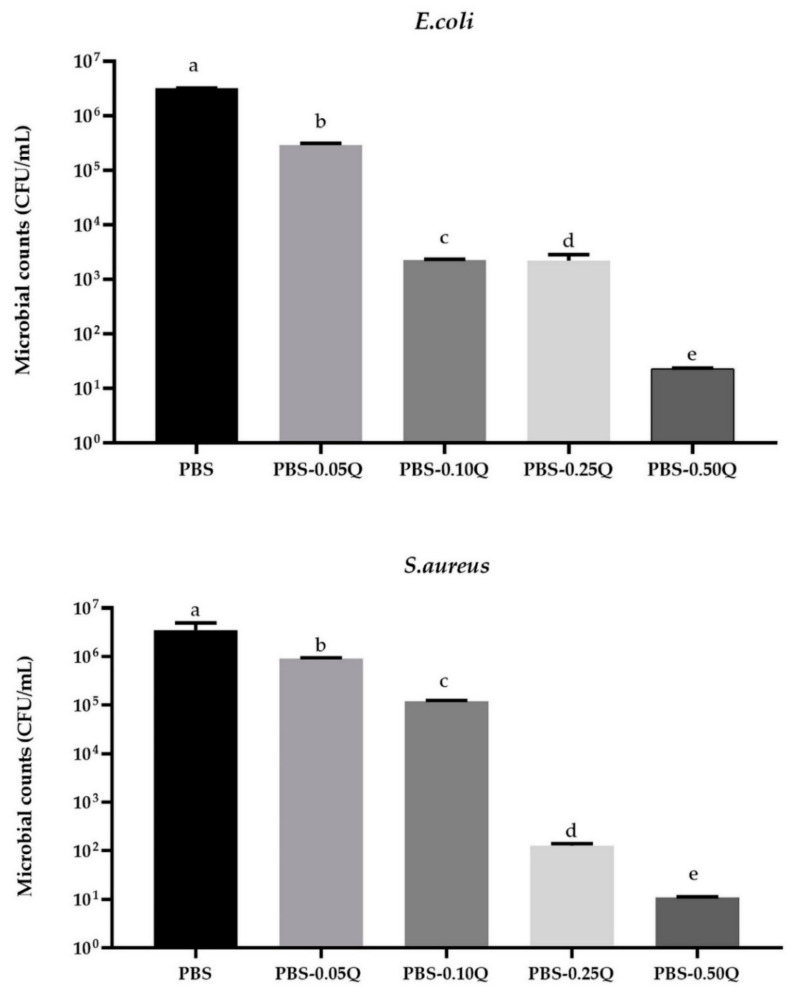
The effect of PBS-based neat and modified films on the viability of *Escherichia coli* and *Staphylococcus aureus* cells.

The antibacterial activity (against Gram-negative *E. coli* and Gram-positive *S. aureus*) of PBS-based film is presented in Figure 1. As can be seen, no reduction of microbial growth was observed for the neat PBS film, which was expected, and is in line with findings of Domínguez-Robles et al. [42] Although for PBS-quercetin film a complete growth inhibition was not reached, a marked reduction in bacterial counts was observed (*p* < 0.05). Thus, it is reasonable to conclude that the films showed some bactericidal activity. For sample PBS-Q0.50, the level of *E. coli* was 2.30 × 10^2^ ± 0.60 CFU/mL, whereas for *S. aureus*, 1.10 × 10^2^ ± 0.36 CFU/mL was noticed. Some antimicrobial activity of quercetin-modified films has already been reported [34,35,38]. Generally, many polyphenolic compounds are reported to exhibit antimicrobial activity [9]. The moderate activity of PBS-quercetin films may be attributed to the low solubility of quercetin in water [21], as well as a relatively low amount of active additive in the films. Gatto et al. [44] reported that quercetin showed weak antimicrobial activity against Gram-positive (*S. aureus*, *Bacillus subtilis*, *Listeria monocytogenes*, *L. ivanovi*, *L. serligeri*) and Gram-negative bacteria (*E. coli*, *Shighella flexneri*, *S. sonnei*, *Salmonella enteridis*, *S. typhimurium*) up to a concentration of 100 µg/mL in aqueous solutions. On the other hand, there are studies that have reported that quercetin is characterized by some antibacterial activity related to a number of factors, such as inhibition of nucleic acids synthesis (inhibition of DNA gyrase), increase of bacterial cell membrane permeability, and dissipation of membrane potential [34]. However, Condat et al. reported the development of photoactivable glycerol-based coatings containing quercetin for antibacterial applications [33]. The coatings were active against *E. coli* and *S. aureus* under light illumination due to the generation of reactive oxygen species, such as singlet oxygen, which were responsible for inhibiting bacteria proliferation. In the present study, as well as in many other studies, bacteria were incubated without light access (laboratory incubator normally used for microbiological tests), thus further investigations should be carried out to determine antimicrobial activity of quercetin-modified PBS films in light conditions.

### 3.3. Color, Opacity and UV–Vis Blocking Effects

**Figure 2:**
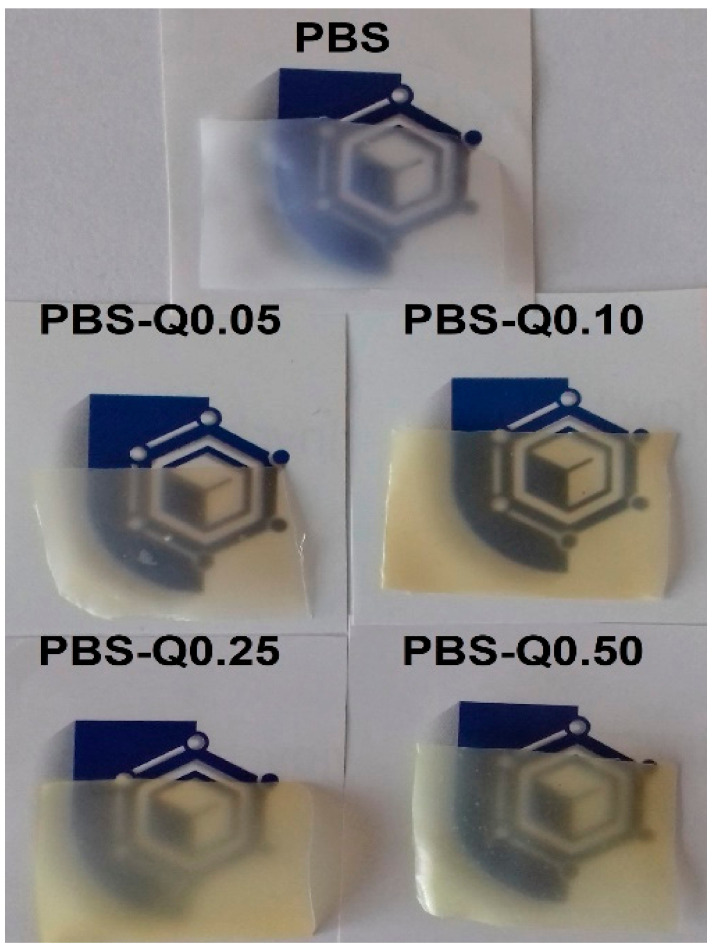
The visual appearance of PBS-based films.

Optical properties are essential to define the ability of films to be applied on a food surface since these can affect the appearance of the coated product [34]. The visual appearance of neat PBS, as well as PBS-quercetin films, is shown in Figure 2, whereas color values are presented in Table 2. Generally, PBS films were characterized with low transparency, and were not fully see-through. The addition of quercetin significantly influenced L*, a*, b*, YI and chroma parameters (*p* < 0.05) due to the dark yellowish coloration of quercetin [34,35]. It was noticed that L* (lightness) considerably decreased with increased quercetin concentration, meaning that the films were darker (*p* < 0.05). Furthermore, a significant decrease of a* (greenness-redness) color coordinate (towards greenness) was observed (*p* < 0.05). Similarly to present results, it has been reported that the addition of quercetin remarkably decreased L* and a* values of chitosan–quercetin [34], and PVA-quercetin [27] films. On the other hand, Giteru et al. reported that a* value increased when quercetin was added to kafirin-based films [35]. Simultaneously, a significant increase of b* (blueness-yellowness) color coordinate (towards yellowness), YI (yellowness index) and chroma were noticed in modified PBS-based films (*p* < 0.05), attributed to the yellow-orange color of quercetin added. For all quercetin-modified films, ΔE values were higher than 1.00 (neat PBS film used as standard), which is considered as perceptible to the human eye [12]. Total color difference (ΔE) values ranged from 15.69 ± 1.77 (PBS-Q0.05) to 25.90 ± 4.27 (PBS-Q0.50), and this parameter is comparable to values reported for carboxymethyl chitosan films modified with quercetin [21]; however, values were lower than reported for PVA-quercetin films [27]. Furthermore, the opacity of the films PBS-Q0.10 (12.72 ± 0.10), PBS-Q0.25 (14.48 ± 0.14) and PBS-Q0.50 (14.96 ± 0.25) increased significantly, in comparison to non-modified PBS film (*p* < 0.05). In the case of the PBS-Q0.50 film, the opacity value was approximately 25% higher than observed for pure PBS films. This increase may make the PBS-quercetin films a barrier to prevent light-induced oxidative deterioration when applied in food products, avoiding nutrient losses, discoloration, and off-flavors [34,36]. The obtained results are in line with the results reported for quercetin-modified chitosan films; however, the opacity of chitosan films increased approximately 89% with the incorporation of quercetin [34].

**Figure 3:**
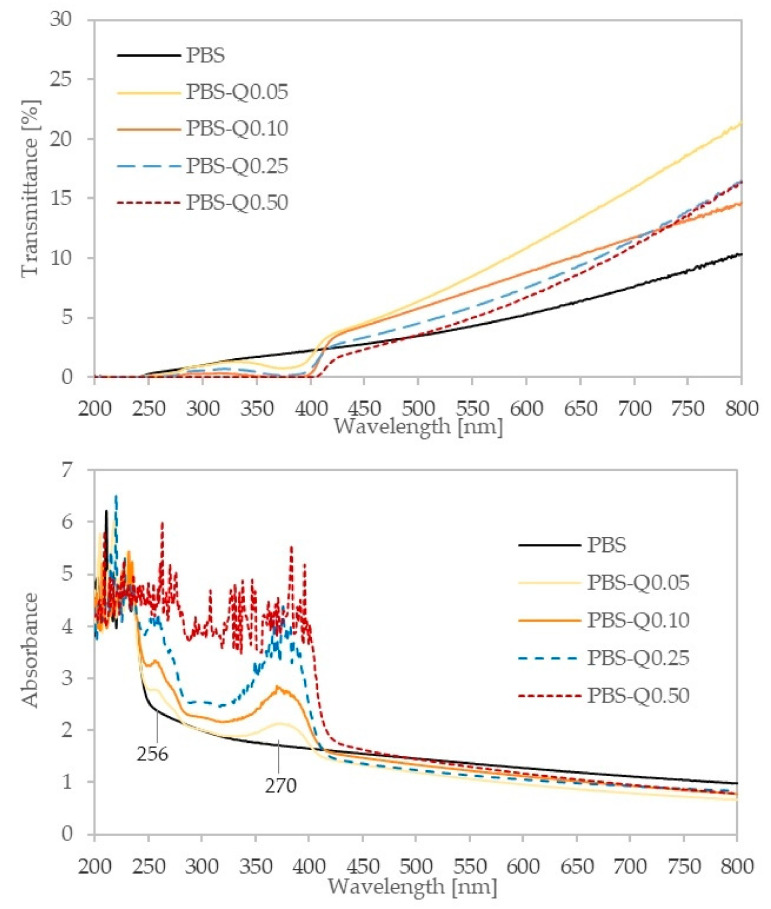
UV–vis spectra of PBS-based neat and modified films.

Barrier properties are the most critical factors for deciding the efficacy of film for packaging applications. Among various barrier properties, UV shielding films have gained excessive attention in recent years since UV light can adversely affect the quality of foods by the generation of free radicals. In this context, UV-blocking additives are the mainstay for good UV-protective films [9,22]. The UV–visible light transmittance and absorbance spectra of the films are shown in Figure 3. All the films were opaque and milky colored due to their transmittance values being quite low. PBS-quercetin films exhibited slightly higher transmittance than the reference sample; however, this parameter decreased with the quercetin content in the films. Quercetin addition improved barrier properties towards UV light in their whole range, and above 0.10 pph, quercetin films provide a total barrier against UV. This attribute could be useful for active packaging applications especially for prolonged shelf-life products that are sensitive to UV light [9]. Comparable results were obtained for PBS films with lignin presence [19] as well as for chitosan–quercetin films [22]. Quercetin as a flavonoid exhibits two major absorption bands in the UV–vis light range: band I at 370 nm assigned to the B-ring absorption (cinnamoyl system), and band II at 256 nm associated with the A ring benzoyl system absorption [45]. As can be seen in Figure 3, increasing absorbance peaks with quercetin content indicates that even small amounts of the flavonoid can effectively block UV rays.

### 3.4. Thickness, Mechanical Properties and Water Vapor Barrier Properties

Table 3 presents thickness, mechanical properties and WVTR values of PBS and PBS-Q films. No statistically significant differences were observed for the thickness of the samples (*p* > 0.05). As can be seen, quercetin presence up to concentration 0.25 pph did not significantly affect the tensile strength of PBS films (*p* > 0.05). On the other hand, for the PBS-Q0.50 sample, a TS approximately 30% lower (8.40 ± 0.50 MPa) than for a neat PBS sample (11.80 ± 2.20 MPa) was observed (*p* < 0.05). The decrease of TS values can be attributed to the change of intramolecular bonding with the addition of quercetin. The addition of a hydrophobic agent to PBS-based films presumably leads to the inner structure of the PBS film becoming discontinued, producing a structure with less chain mobility and, consequently, with less resistance to fracture [23,24,27,34,46]. Regarding elongation at break (EB), a decreasing tendency with increasing quercetin content was noticed (*p* < 0.05). EB of PBS-Q0.50 (81.00 ± 9.20%) was approximately 54% lower than EB of neat PBS film (155.00 ± 32.10%). Similarly, a decrease of YM was observed (*p* < 0.05). A comparable influence of quercetin on mechanical properties was reported for carboxymethyl chitosan-quercetin [21], PVA-quercetin [27] as well as for PBS/tapioca starch films modified with empty fruit bunch fibers [18] as well as for PBS films with graphene oxide [47]. In Wan and Chen’s work [47], mechanical properties of PBS/graphene oxide (GO) composites slightly decreased when 0.3% and 0.5% of GO were added and the improvement of tensile properties were obtained for a higher amount of GO. On the other hand, Braga et al. reported two-fold higher EB values for PVC films with 0.4% quercetin compared to non-modified PVC [23]. Therefore, the mechanical properties of modified PBS films can be dependent on the concentration of the additive. Sahoo et al. reported that when lignin was added at levels 30% and 50%, the TS of the films decreased. Conversely, when the level of lignin was 65%, the TS of the films increased when compared to non-modified PBS films [19]. It should be emphasized that the absence of cross-linked agent or plasticizer is one of the important key factors causing low mechanical properties of the films [18]. The application of plasticizer helps to reduce the brittleness of polymers, consequently having higher flexibility of specimens. Therefore, it can be concluded that the addition of quercetin into PBS-reduced film’s mechanical performances.

The water vapor transmission rate is one of the most important parameters of packaging materials since it determines the ability of the films to interact with water to promote protection against the dehydration process or rehydration of the food matrix [34]. As can be seen in Table 3, some fluctuations occurred in the WVTR values of pure and modified PBS; however, no statistically significant differences were observed (*p* > 0.05). The values obtained in this study are higher than reported by Xu et al. [3]. Souza et al. reported that the addition of quercetin to chitosan films did not lead to the change of water vapor permeability [34]. Similarly, Giteru et al. found that quercetin did not affect WVTR of kafirin films [35]. On the other hand, Han et al. reported that the effect of quercetin on water vapor permeability of high density polyethylene (HDPE) and ethylene-vinyl acetate (EVA) copolymer was dependent on concentration [39]. When the quercetin level in films was 0.74%, an increase of WVTR value was noticed, attributed to potential defects of the polymeric matrix. When the addition was 1.23%, the WVTR significantly decreased due the hydrophobicity of quercetin [39].

### 3.5. Moisture Content, Moisture Sorption, Water Sorption and Migration of Quercetin into Model Food Liquid Systems

The moisture content of the samples is presented in Table 4. As can be seen, the addition of quercetin caused a significant decrease in moisture content (*p* < 0.05). When considering moisture sorption, a similar trend was noticed with increased quercetin content (*p* < 0.05). However, the samples stored in 80% RH showed higher moisture sorption values than samples stored in 50% RH (*p* < 0.05). Similarly, water sorption of samples modified with quercetin was found to be lower than that of neat PBS (*p* < 0.05). Those observations can be linked with hydrophobicity of quercetin, which hindered formation of polymer–water hydrogen bonds, resulting in a limited ability to absorb moisture and water [39,41].

**Figure 4:**
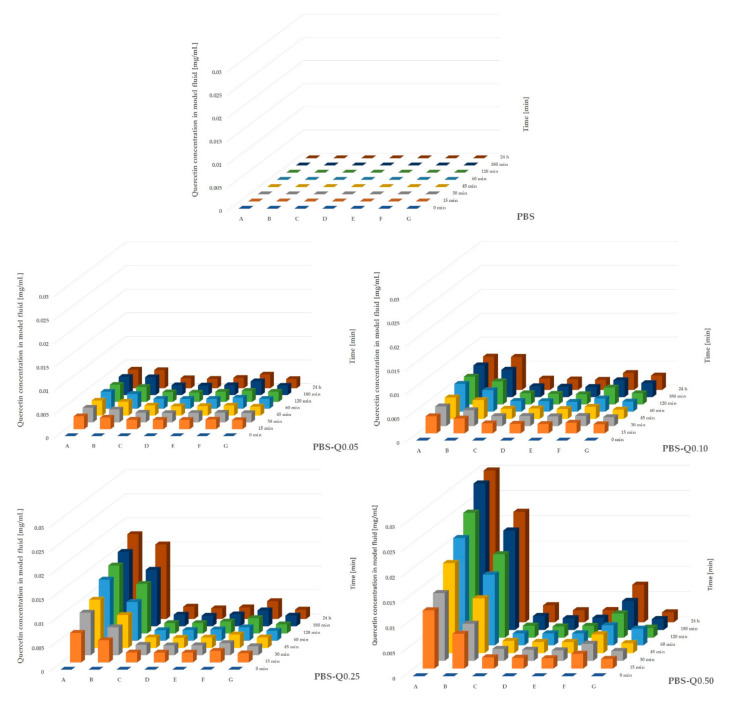
Migration of quercetin into model fluids: A—96% ethanol; B—50% ethanol; C—20% ethanol; D—10% ethanol; E—3% acetic acid; F—0.01M NaOH; G—distilled water.

The quercetin migration tests were performed using several model liquid food systems: distilled water, 96% ethanol, 50% ethanol, 20% ethanol, 10% ethanol, 3% acetic acid, and 0.01 M NaOH. As can be seen in Figure 4, the highest concentrations of quercetin were noticed when 96% ethanol was used, followed by 50% ethanol and 0.01 M NaOH. On the other hand, lower concentrations were noticed for 20% ethanol, 10% ethanol, 3% acetic acid, and distilled water. It was also observed that the highest concentration of released quercetin for all modified PBS films was detected after 24 h. Generally, good solubility of quercetin in organic solvents (such as ethanol) and poor solubility in water are reported [48,49]. However, the solubility of quercetin increases in alkaline aqueous solutions conditions due to chemical modification consisting of an autoxidation that mainly affects the C-ring [48,49]. This phenomenon can be of particular importance when some food types (meat, fish) lose their freshness and start to decay. Microorganisms can act on food matrix proteins via proteolytic activity and transform them into smaller compounds, such as free amino acids. These amino acids undergo oxidative deamination, decarboxylation, and desulfurization, resulting in NH_3_, CO_2_, and H_2_S. Moreover, another indicator of the microbial decay of proteins in food is high levels of total volatile basic nitrogen (TVBN), such as ammonia, dimethylamine (DMA), trimethylamine (TMA), and biogenic amines, such as histamine and tryptamine [50,51,52].

### 3.6. FT-IR Results

**Figure 5:**
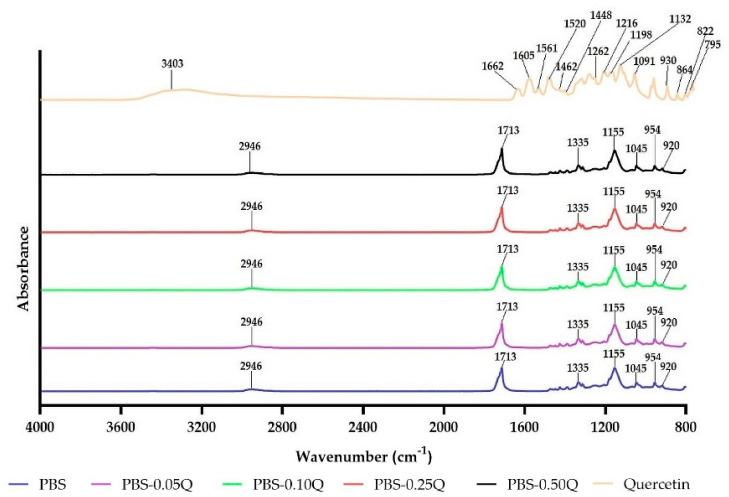
Fourier-transform infrared (FT-IR) spectra of PBS-based films.

The FT-IR spectra of quercetin, neat PBS and PBS-quercetin films are presented in Figure 5. For quercetin, FT-IR spectrum peaks at 3403 and 1448 cm^−1^ were detected, derived from -OH groups. The C=O aryl stretching absorption was observed at 1662 cm^−1^. The peaks at 1605, 15561 and 1520 cm^−1^ can be attributed to the C=C stretching vibration of the aromatic ring [22]. The signals from C–O bonds were found at 1462 and 1132 cm^−1^. The in-plane bending bands were noticed at 930, 822 and 678 cm^−1^. Bands detected at 1262, 1198 and 1168 cm^−1^ can be attributed to the C–O stretching in the aryl ester ring, the C–O stretching in phenol, and the C–CO–C stretch and bending in the ketone, respectively. Moreover, the signals from the C–OH deformation and -C–OH stretch vibration were observed at 1214 and 1091 cm^−1^. Some minor signals resulted from substituted benzene were noticed at 864, 795 and 700 cm^−1^ [22,27]. Pure PBS and PBS-quercetin films showed characteristic peaks for the ester group (O=C–O) located at 1717 cm^−1^ and the carbonyl group (C=O) observed at 1713 cm^−1^. Moreover, signals from the C–O group at 1155–1145 cm^−1^ (centered at 1335 cm^−1^) and the C–H group at 2946 cm^−1^ were noticed [10].

No qualitative and quantitative changes were observed as a result of quercetin addition to PBS. This might be attributed to low quercetin content, as quercetin-derived signals were overlapped by derived from PBS. This suggests that hydrogen bonding between quercetin and PBS was not formed. On the contrary, Bai et al. reported that the addition of quercetin to more hydrophilic carboxymethylated chitosan (CMCS) films generated new bands, and the intensities of these new bands increased with the enhancement of quercetin content; however, quercetin concentration was in the range of 2.5–7.5% on CMCS [21]. It should be emphasized that CMCS (contrary to PBS) contains –OH and –COOH groups, which can participate in the formation of hydrogen bonds.

### 3.7. Results of PBS Films Thermal Characterization

**Figure 6:**
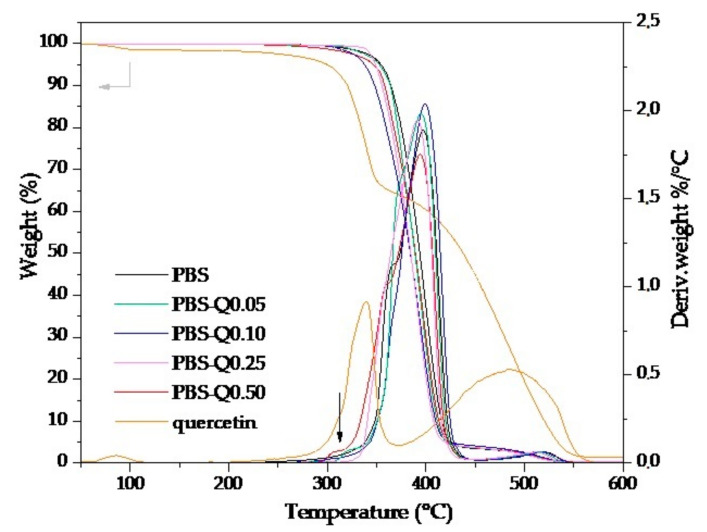
TGA and DTA curves of quercetin and PBS-based films.

The TGA curves depicting the thermal stability of PBS films and the active components are illustrated in Figure 6. As can be seen in the case of quercetin, there was a three-step weight loss; the first one at approximately 90 °C can be attributed to moisture evaporation, the second one at maximum DTA peak 340 °C (33% of weight loss) corresponds to the decomposition of the central C ring or with the loss of one of the two A and B di-hydroxylated rings [53], and the third peak at 485 °C is related to the decomposition of organic matter. There was no significant difference between PBS films and their analogs with quercetin. The maximum peak of PBS DTA curves was noticed at 399 °C, and the temperature range for PBS-Q films was 392–397 °C. The barely noticeable DTA peak at approximately 310 °C for PBS-Q0.50 could be related to quercetin degradation in the polymer matrix.

**Figure 7:**
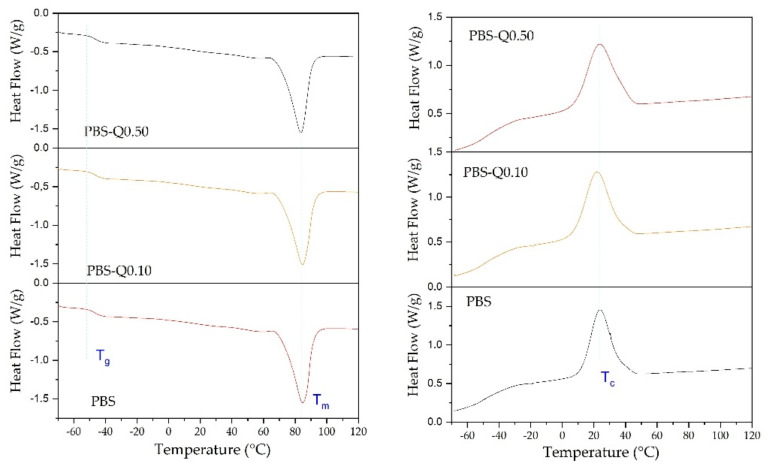
DSC curves for PBS-based films in heating (**left**) and cooling (**right**) run; T_g_—glass transition, T_m_—melting transition, T_c_—crystalization transition.

DSC analysis was performed to study the influence of quercetin on the PBS phase transitions. As can be noticed from Figure 7, the studied PBS and PBS with two different Q contents in the heating run exhibited glass transition at −50.5 °C, melting transition at approximately 84 °C and, in the cooling run, crystallization transition at ca. 42 °C. The presence of an active substance did not affect the thermal properties of the PBS matrix. Since quercetin was added in small amounts, it did not affect the PBS thermal properties. Similar results were obtained in the work of Ali and Mohan, where properties of PBS with carbon nanotubes (CN) were studied [54]. An impact of CN on thermal transitions occurred for 3% and higher of the filler. Low glass transition of the samples could be an advantage for packaging materials stored as frozen food products. The lack of impact of Q addition into PBS on their thermal properties correlates with FT-IR results that indicated the absence of interaction between active additive and polymer matrix.

## 4. Conclusions

The present work confirmed that quercetin can be successfully added to PBS-based films, acting as a functional additive. The modification with quercetin had a marked effect on the mechanical properties of the PBS films. Moreover, results showed that PBS-quercetin films may be used as active packaging for food products as they showed a UV-blocking effect, moderate antibacterial and excellent antioxidant activity in vitro. It is, therefore, suggested that PBS–quercetin films and coatings may be used as active packaging materials, contributing to food preservation and shelf-life extension. However, further tests of the influence of the developed materials on various food products should be carried out to determine their suitability in food technology.

## Figures and Tables

**Table 1:** Reducing power (RP) and free radical scavenging activity of PBS-based films.

Sample	RP (700 nm)	DPPH (%)	ABTS (%)	O_2_^−^ (%)
PBS	0.41 ± 0.28 ^a^	0.21 ± 0.00 ^a^	1.61 ± 0.31 ^a^	0.21 ± 0.01 ^a^
PBS-Q0.05	0.88 ± 0.03 ^b^	38.79 ± 0.07 ^b^	25.51 ± 0.82 ^b^	15.21 ± 0.12 ^b^
PBS-Q0.10	0.96 ± 0.05 ^b^	42.77 ± 0.07 ^c^	48.84 ± 3.43 ^c^	16.69 ± 0.23 ^c^
PBS-Q0.25	1.68 ± 0.13 ^c^	80.12 ± 0.00 ^d^	79.16 ± 5.74 ^d^	29.23 ± 0.35 ^d^
PBS-Q0.50	2.08 ± 0.01 ^d^	80.90 ± 0.07 ^e^	99.07 ± 0.28 ^e^	38.06 ± 0.11 ^e^

Values are means ± standard deviation. Means with a–e letters in columns are significantly different at *p* < 0.05.

**Table 2:** Color, total color difference (ΔE), yellowness index (YI), opacity and chroma (C) of PBS-based films.

Sample	L*	a*	b*	ΔE	YI	Opacity	C
PBS	85.34 ± 1.28 ^a^	−0.33 ± 0.19 ^a^	2.86 ± 0.51 ^a^	used as standard	4.78 ± 0.84 ^a^	11.94 ± 1.07 ^a^	2.89 ± 0.48 ^a^
PBS-Q0.05	80.58 ± 3.69 ^ab^	−0.19 ± 0.47 ^a^	18.38 ± 1.86 ^b^	15.69 ± 1.77 ^a^	31.40 ± 3.13 ^b^	12.40 ± 2.04 ^a^	18.39 ± 1.85 ^b^
PBS-Q0.10	83.61 ± 1.38 ^a^	−0.80 ± 0.17 ^a^	19.53 ± 4.70 ^b^	17.92 ± 3.60 ^b^	34.44 ± 7.43 ^b^	12.72 ± 0.10 ^b^	19.55 ± 4.69 ^b^
PBS-Q0.25	79.59 ± 4.63 ^b^	−1.48 ± 0.21 ^b^	21.89 ± 2.28 ^bc^	20.46 ± 1.47 ^c^	39.24 ± 2.80 ^bc^	14.48 ± 0.14 ^c^	21.93 ± 2.28 ^bc^
PBS-Q0.50	76.35 ± 4.97 ^b^	−2.66 ± 0.37 ^c^	26.34 ±5.49 ^c^	25.90 ± 4.27 ^d^	49.19 ± 9.52 ^c^	14.96 ± 0.25 ^d^	26.45 ± 5.47 ^c^

Values are means ± standard deviation. Means with a–e letters in columns are significantly different at *p* < 0.05.

**Table 3:** Tensile strength (TS), elongation at break (EB), Young’s modulus (YM), thickness and water vapor transmission rate (WVTR) of PBS-based films.

Sample	TS (MPa)	EB (%)	YM (MPa)	Thickness (mm)	WVTR (g/m^2^ × Day)
**PBS**	11.80 ± 2.20 ^a^	155.00 ± 32.10 ^a^	144.00 ± 19.00 ^a^	0.18 ± 0.02 ^a^	60.27 ± 5.08 ^a^
**PBS-Q0.05**	11.90 ± 2.90 ^a^	140.00 ± 48.40 ^ab^	147.00 ± 17.00 ^a^	0.18 ± 0.04 ^a^	63.02 ± 3.44 ^a^
**PBS-Q0.10**	12.10 ± 1.10 ^a^	114.00 ± 14.21 ^abc^	119.00 ± 14.30 ^b^	0.17 ± 0.05 ^a^	62.79 ± 4.09 ^a^
**PBS-Q0.25**	11.90 ± 0.70 ^a^	94.00 ± 30.50 ^cb^	128.00 ± 13.50 ^b^	0.16 ± 0.01 ^a^	59.21 ± 7.13 ^a^
**PBS-Q0.50**	8.40 ± 2.50 ^a^	81.00 ± 9.20 ^c^	100.00 ± 16.50 ^b^	0.19 ± 0.04 ^a^	59.41 ± 8.29 ^a^

Values are means ± standard deviation. Means with a–e letters in columns are significantly different at *p* < 0.05.

**Table 4:** Moisture content, moisture sorption (MS) and water sorption (WS) of PBS-based films.

Sample	MC (%)	MS (50% RH) (%)	MS (80%) (%)	WS (%)
PBS	1.58 ± 0.11 ^a^	1.21 ± 0.12 ^Aa^	1.26 ± 0.12 ^Ba^	1.61 ± 0.13 ^a^
PBS-Q0.05	1.21 ± 0.07 ^b^	1.17 ± 0.06 ^Aa^	1.20 ± 0.15 ^Ba^	1.17 ± 0.15 ^b^
PBS-Q0.10	0.77 ± 0.03 ^c^	0.68 ± 0.13 ^Ab^	0.84 ± 0.11 ^Bb^	0.77 ± 0.09 ^c^
PBS-Q0.25	0.76 ± 0.07 ^c^	0.61 ± 0.13 ^Ab^	0.82 ± 0.17 ^Bb^	0.77 ± 0.06 ^c^
PBS-Q0.50	0.64 ± 0.10 ^c^	0.51 ± 0.08 ^Ab^	0.74 ± 0.09 ^Bb^	0.64 ± 0.13 ^c^

Values are means ± standard deviation. Means with a–e letters in columns are significantly different at *p* < 0.05. Means with A–E letters in rows are significantly different at *p* < 0.05.

## Data Availability

The data presented in this study are available on request from the corresponding author.
